# Characterizing the contribution of alcohol use towards neuropsychological profiles in mild cognitive impairment

**DOI:** 10.1002/alz70857_100017

**Published:** 2025-12-24

**Authors:** Ari Cuperfain, Sara Pishdadian, Malcolm A Binns, Sandra E. Black, Howard Chertkow, Morris Freedman, Janine Louis, Clement Ma, Mario Masellis, Paula M McLaughlin, Joel Ramirez, David F. Tang‐Wai, Carmela Tartaglia, Sanjeev Kumar, CCNA COMPASS‐ND Group, ONDRI Group

**Affiliations:** ^1^ University of Toronto Temerty Faculty of Medicine, Toronto, ON, Canada; ^2^ Centre for Addiction and Mental Health, Toronto, ON, Canada; ^3^ Rotman Research Institute, Baycrest Health Sciences, Toronto, ON, Canada; ^4^ Sunnybrook Health Sciences Centre, University of Toronto, Toronto, ON, Canada; ^5^ Baycrest Health Sciences, Toronto, ON, Canada; ^6^ Sunnybrook Health Sciences Centre, Toronto, ON, Canada; ^7^ Nova Scotia Health, Halifax, NS, Canada; ^8^ Krembil Brain Institute, University Health Network (UHN), Toronto, ON, Canada

## Abstract

**Background:**

Excessive alcohol use is known to increase the risk of dementia, however, how alcohol specifically effects cognition in patients with neurocognitive disorders is unclear. In this study, we characterized the effects of excessive alcohol use on cognitive domains in those with mild cognitive impairment (MCI).

**Method:**

Two multicenter cohorts, the Comprehensive Assessment of Neurodegeneration and Dementia and the Ontario Neurodegenerative Disease Research Initiative, provided data for this study. Participants were diagnosed with MCI due to Alzheimer's disease (AD‐MCI) or cerebro‐vascular disease (V‐MCI), and were categorized based on their current and past alcohol use into ‘zero’, ‘low‐medium’ (less than 1 to 7 standard drinks/week), or ‘high’ (>7 standard drinks/week) alcohol use groups. We generated age‐corrected composite cognitive domain scores for Processing Speed, Memory Encoding, Recall and Recognition, Executive Control, Executive Function, Visuoperceptual, and Language domains, and compared them among the three groups while controlling for sex, pre‐morbid intelligence, and diagnosis.

**Result:**

The zero alcohol group included 157 participants (females = 48.4%; V‐MCI = 52.2%) with mean (SD) age 71.26 (6.93) years, the low‐medium alcohol group included 213 participants (females = 42.2%; V‐MCI = 44.1%) with mean (SD) age 71.64 (7.77) years, and the high alcohol group included 73 participants (females = 20.5%; V‐MCI = 50.1%) with mean (SD) age 72.23 (7.51) years. The groups differed only in Processing Speed (F(2,407) = 3.298, *p* =  0.038), Memory Encoding (F(2,394) = 4.689, *p* =  0.010) and Recall (F(2,403) = 3.997, *p* =  0.019). Pairwise comparisons revealed a significant difference between the low‐medium and high alcohol groups in both Memory Encoding (*p* = 0.019) and Recall (*p* = 0.018), with participants in the high alcohol group demonstrating lower performance. There were no differences between the zero alcohol group and other groups. There were no pairwise differences for Processing Speed.

**Conclusion:**

In participants with AD‐MCI or V‐MCI, excessive alcohol use is associated with worse memory encoding and retrieval, but not retention. This is consistent with a more dysexecutive memory profile than a typical amnestic cognitive profile. Future studies should investigate underlying mechanisms for these deficits, and their impact on prognosis of the illness.

